# CPPLS-MLP: a method for constructing cell–cell communication networks and identifying related highly variable genes based on single-cell sequencing and spatial transcriptomics data

**DOI:** 10.1093/bib/bbae198

**Published:** 2024-04-27

**Authors:** Tianjiao Zhang, Zhenao Wu, Liangyu Li, Jixiang Ren, Ziheng Zhang, Guohua Wang

**Affiliations:** College of Computer and Control Engineering, Northeast Forestry University Harbin, 150040, China; College of Computer and Control Engineering, Northeast Forestry University Harbin, 150040, China; College of Computer and Control Engineering, Northeast Forestry University Harbin, 150040, China; College of Computer and Control Engineering, Northeast Forestry University Harbin, 150040, China; College of Computer and Control Engineering, Northeast Forestry University Harbin, 150040, China; College of Computer and Control Engineering, Northeast Forestry University Harbin, 150040, China; Faculty of Computing, Harbin Institute of Technology Harbin, 150001, China

**Keywords:** cell communication, highly variable genes, scRNA-seq, ST-seq

## Abstract

In the growth and development of multicellular organisms, the immune processes of the immune system and the maintenance of the organism’s internal environment, cell communication plays a crucial role. It exerts a significant influence on regulating internal cellular states such as gene expression and cell functionality. Currently, the mainstream methods for studying intercellular communication are focused on exploring the ligand–receptor–transcription factor and ligand–receptor–subunit scales. However, there is relatively limited research on the association between intercellular communication and highly variable genes (HVGs). As some HVGs are closely related to cell communication, accurately identifying these HVGs can enhance the accuracy of constructing cell communication networks. The rapid development of single-cell sequencing (scRNA-seq) and spatial transcriptomics technologies provides a data foundation for exploring the relationship between intercellular communication and HVGs. Therefore, we propose CPPLS-MLP, which can identify HVGs closely related to intercellular communication and further analyze the impact of Multiple Input Multiple Output cellular communication on the differential expression of these HVGs. By comparing with the commonly used method CCPLS for constructing intercellular communication networks, we validated the superior performance of our method in identifying cell-type-specific HVGs and effectively analyzing the influence of neighboring cell types on HVG expression regulation. Source codes for the CPPLS_MLP R, python packages and the related scripts are available at ‘CPPLS_MLP Github [https://github.com/wuzhenao/CPPLS-MLP]’.

## INTRODUCTION

Cell communication plays a pivotal role in multicellular organisms, driving cell differentiation, orchestrating the harmonious functioning of tissues and organs and regulating both beneficial and detrimental immune responses in diseases [1]. The rise of single-cell and transcriptomic technologies has provided crucial technical support and foundational data for comprehending the diverse cellular landscape within tissues and organs [2]. These advancements have also empowered detailed exploration of molecular interactions and information exchange at the molecular level among distinct cell populations. In the realm of tumor research, the expression of programmed cell death-Ligand 1 (PD-L1) protein within tumor cells, binding with programmed death 1 (PD-1) protein on T cells, modulates T-cell gene expression patterns, attenuating the immune response against tumor growth [3]. Disrupting this intercellular communication via PD-1 pathway inhibitors effectively curtails tumor cell proliferation. To unravel and manage intricate multicellular systems, dissecting intercellular information exchange stands as a potent approach for uncovering the regulatory mechanisms governing gene expression [4–6].

In recent years, significant advancements have been made in methodologies to predict ligand–receptor interactions (LRIs) in single-cell RNA sequencing (scRNA-seq) and spatial transcriptomics (ST) data [7–9]. These approaches have shed light on the intricate communication processes between cells. Notably, Ramilowski and colleagues pioneered the construction of a meticulously curated dataset comprising 2422 LRIs [10]. This dataset has become a cornerstone in studying intercellular information exchange. Advances in databases and statistical tools have enhanced our ability to infer communication mechanisms between diverse cell types. Current strategies primarily revolve around two approaches. The first leverages highly co-expressed ligands and receptors as potential mediators of cell communication. Integration of extensive ligand and receptor information enables signaling inference from signal senders to receivers, exemplified by tools like CellphoneDB [11–13]. The second approach focuses on downstream targets activated in receptors due to LRIs. This strategy enriches and assesses ligand–receptor–downstream target signaling networks and is implemented in methods such as NicheNet and Cellchat [7–9, 14]. Despite these advancements, our understanding of the relationship between HVGs and intercellular communication remains limited.

scRNA-seq and ST technologies have been extensively applied to delve into the complexities of multicellular systems. These methods have unveiled highly variable gene expression within the same cell types, a variation crucial for normal growth, development and disease states [11, 15–18].

A comprehensive understanding of the relationship between HVGs and intercellular communication holds significant implications for unraveling the intricate mechanisms underlying cellular interactions and exploring disease progression [19]. Yet, research on HVG expression has predominantly focused on computational tools utilizing scRNA-seq data to infer potential cell–cell communications. These tools typically deduce intercellular signaling by comparing the expression levels of ligand and receptor genes across different cell types [20–22]. However, these methods come with inherent limitations, including incomplete knowledge of ligand–receptor pairs, potential crosstalk between ligands and receptors and the challenge of capturing cellular spatial contexts.

Gene expression in cells is influenced by the spatial arrangement and configuration of neighboring cells, akin to an MIMO system [23, 24]. Recently, several methods have emerged to decipher the spatial communication mechanisms between cells, with some taking into account the MIMO framework. For instance, Giotto utilizes the concept of ‘preferred cell neighbors’ among different cell types in single-cell ST datasets. It employs enrichment testing to assess the likelihood of specific cell–cell interactions in adjacent co-expressing cells, thereby inferring spatial communication modes between cells [25]. CCPLS, on the other hand, applies single-cell and ST datasets. It utilizes Partial Least Squares (PLS) regression to model the linear relationship between HVG intercellular differential expression and cell spatial coordinates. This approach deduces the impact of intercellular communication among different cell types on HVG expression [26]. However, Giotto and CCPLS are limited to inferring the possibility of proximal co-expression in single-cell and ST data (rather than the entire cellular environment) and the linear relationship between individual gene expression and cell coordinates. Moreover, these methods focus on multiple inputs (MIs) rather than multiple outputs (MIMO) of intercellular communication. Notably, these methods lack a standardized classification criterion to determine whether a gene is associated with cell communication. To date, there is still a significant challenge in establishing metrics to gauge whether genes are related to cell communication. Furthermore, the challenge extends to fitting continuous, discrete and combined signals, representing multiple dependent and independent variables, in constructing MIMO networks for intercellular communication. These challenges pose significant hurdles in decoding the spatial cellular dynamics of potential disease pathologies.

To address this challenge, we propose CPPLS-MLP, a method that integrates Constrained Partial Least Squares (CPPLS) [27, 28] and Multilayer Perceptron (MLP) [29, 30] techniques, for the construction of MIMO cell communication networks and gene classification. CPPLS-MLP combines gene expression data and cell spatial coordinates, modeling the impact of intercellular communication among multiple cell types on HVGs. It introduces a novel criterion for genes associated with cell communication and utilizes MLP for gene classification under this criterion. CPPLS-MLP demonstrates superior stability in the selection of HVGs from the dataset compared to other methods. The kernel functions employed in CPPLS-MLP highlight the uniqueness of HVGs within each cell type, surpassing other methodologies. Applied to Seq-scope and seqFISH+ datasets, CPPLS-MLP unveils the regulatory role of intercellular communication across various cell types on HVG expression. Furthermore, it classifies these HVGs according to the new criteria. These results underscore CPPLS-MLP as a novel approach to comprehend how spatial intercellular communication within tissues impacts HVG expression. Importantly, it proves valuable in unraveling cell–cell communication at single-cell and ST resolutions, showcasing its practical utility in this domain.

## METHODS

Constrained Partial Least Squares introduces additional constraints on top of PLS to control model properties and behaviors. Apart from minimizing the residual sum of squares, it imposes extra constraints such as regularization on regression coefficients and relationships among regression coefficients. Multilayer Perceptron is a feedforward neural network model consisting of multiple neurons, including input layers, multiple hidden layers and output layers. Each neuron connects with all neurons from the previous layer via weights, and through learning these weights, it approximates the output of a function, achieving complex mappings from inputs to outputs.

The nonlinear modeling capability of MLP enables it to capture complex interactions in gene expression data and genetic networks, revealing hidden patterns and relationships. Its multi-layer structure allows for the extraction of advanced features layer by layer, uncovering deeper biological insights. In contrast, the CNN and other graph network methods are better suited for data with grid-like structures, such as images, while the high-dimensional and unstructured nature of gene expression data may limit their effectiveness in feature extraction.

### Cell communication network construction

To process single-cell and spatial transcriptomic data, let *W* represent the number of genes, *Z* denote the number of cells and *A* indicate the number of cell types. The gene expression matrix U∈${V}^{Z\ast W}$ contains each cell *c* (1 ≤ *c* ≤ Z) and the expression values ${u}_{c,w}$ for each gene [31, 32]. The coordinate matrix O∈${V}^{Z\ast 2}$ includes the two-dimensional spatial coordinates $({O}_{c,1}$, ${O}_{c,2})$ (1 ≤ *c* ≤ *Z*) for each cell. The cell type label vector D = {${d}_c$|${d}_c$ ∈ {1, …,*A*}} consists of A unique cell type labels ${d}_c$. We assume the presence of specific HVGs, denoted as ${h}^a$ within each cell type *a*, which includes the expression matrix ${U}^a$ for cells c(1 ≤ c≤${Z}^a$) of cell type a. Based on this data, a linear model is established:


$$ {u}_{c,h}^{(a)}=\sum_b\kern0.1em {f}_{c,b}^{(a)}{w}_{b,h}^{(a)}+{\epsilon}_{c,h}, $$

Here, h∈${h}^a$, ${w}_{b,h}^{(a)}$ denotes the coefficient, ${f}_{c,b}^{(a)}$ represents the score of neighboring cell type and ${\varepsilon}_{c,h}$ is the residual term. CPPLS-MLP assumes that for each HVG, neighboring cell type *b* acts uniquely on cell type *a*. Therefore, its objective is to estimate the direction and magnitude of cell–cell communication regulation using the coefficients ${w}_{b,h}^{(a)}$.

The coefficient represented by *W* signifies the relationship between genes and adjacent cells after the input data passes through the model. The magnitude of the coefficient reflects the degree of regulation exerted by adjacent cells on this HVG. On the other hand, *f* denotes the regulatory direction of adjacent cells on this HVG. Its value can be positive or negative, where positive indicates upregulation and negative signifies downregulation of expression.

Here, ${u}_{c,h}^{(a)}$ (*h* = 1, …, *H*) represents the preprocessed expression values of HVGs, ‘H’ represents processed HVGs represented by a number from 1 to *H*. CPPLS-MLP divides the gene expression matrix U into A matrices, each corresponding to a specific cell type. ${U}^{\prime (a)}$∈ *U* represents the expression values within cell type *a*, where genes with zero expression across all cells of cell type *a* are removed, and the remaining genes’ z-scores are normalized. CPPLS-MLP identifies specific HVG ${h}^a$ within cell type *a*.

In the equation, ${f}_{c,b}^{(a)}$ represents the calculated score for adjacent cell types for each type *a*. CPPLS-MLP computes the raw adjacent cell type scores ${f}_{c,b}^{\prime (a)}$ from the input coordinate matrix O and cell type label vector D.


$$ {f}_{c,b}^{\prime (a)}=\sum_m{s}_{n,b,m}\log 10\left(\frac{distm,n}{dist0}\right), $$

where ${s}_{n,b,m}$ represents a binary value determining whether cell *n* belonging to cell type label ${d}_n$ is also of cell type *b*(1 ≤ *b* ≤ *Z*，*m* ≠ *n*), ${dist}_{m,n}$ represents the Euclidean distance calculation between cells *m* and *n* based on their coordinates and $\mathrm{d} ist0$ is set as the minimum Euclidean distance among all pairwise cell combinations in the coordinate matrix O. Finally, z-score transformation is applied to the raw adjacent cell type scores calculated for each cell type *b*, resulting in the matrix ${U}^a$ of adjacent cell type scores.

### CPPLS-MLP regression modeling

For each cell type *a*, CPPLS-MLP performs CPPLS regression modeling. CPPLS-MLP leverages CPPLS regression with two major advantages: (1) its capability to handle the ‘small N, large P’ problem, a characteristic of ST [33, 34] and (2) its ability to handle continuous, discrete and combinatorial data, reflecting the cooperative maintenance of all genes and cells within the internal environment homeostasis.

Firstly, we can consider the initial step as a process composed of the evolution of external relationships (measured by F and U). These two data matrices are decomposed into latent variables and residual matrices. These submatrices can be represented as products of scores and loadings, which are then recombined into separate matrices, as illustrated below:


$$ F=X{P}^T+I, $$

$$ U=Y{Q}^T+J, $$

Here, X∈${R}^{Z\ast C}$ and Y∈${R}^{Z\ast C}$ represent the score matrices of F and U matrices, respectively. ${P}^{D\ast C}$ and ${Q}^{D\ast C}$ are the loading matrices of F and U matrices, where C denotes the number of components in the CPPLS regression. The matrices I∈${R}^{Z\ast D}$ and J∈${R}^{Z\ast H}$ correspond to the residuals of the CPPLS regression model.

The second step is to calculate the supplementary score matrix,


$$ {X}^{\prime }= FY, $$

$$ {Y}^{\prime }= UX, $$

*X*′ and *Y′* in the formula represent the supplementary score matrices of *F* and *U*, respectively.

The third step is to fit the intrinsic linear relationship between *X* and *Y*,


$$ Y= XK+H, $$

In this equation, *K*∈${R}^{C\ast C}$ represents a diagonal matrix, and *H*∈${R}^{Z\ast C}$ represents the residual matrix. Finally, the result of the CPPLS regression model can be expressed as follows:


$$ U= FW+G, $$

W∈${R}^{D\ast H}$ in the formula represents the coefficient matrix,


$$ W={F}^TY{\left(X^\prime FFY\right)}^{-1}X{^\prime}^TU. $$

G∈${R}^{Z\ast H}$is the residual matrix.

For each cell type *a*, after performing CPPLS regression modeling, CPPLS-MLP obtains a matrix ${W}^{(a)}$ composed of coefficients ${w}_{b,h}^{(a)}$(1 ≤ b ≤ A, h∈$\left\{{h}^{(a)}\right\}$). Each coefficient ${w}_{b,h}^{(a)}$ is the sum of all genes${w}_{b,h}^{(a)}={\sum}_c{w}_{b,h,c}^{(a)}$ in the corresponding block *c*. Finally, the direction and magnitude of cell–cell communication regulation are estimated based on the coefficient matrix [35]. To prevent our model from developing a preference for certain data, we input gene expression data and spatial coordinate information into the model and divide these data into 10 subsets of approximately equal size. By repeatedly using different subsets as the test set and the remaining nine as the training set, we can reduce bias introduced by unreasonable dataset division. In each round of cross-validation, the model is trained and validated on different subsets of data, effectively preventing the model from overfitting.

### Filtering of coefficients

CPPLS-MLP filters the coefficient ${w}_{b,h}^{(a)}$ twice. The first filtering step involves performing a *t*-test on the factor loadings ${w}_{b,h,c}^{(a)}$ from each block c obtained in the CPPLS regression modeling. This test is applied to derive the *P*-values corresponding to the Pearson correlation coefficients [36]. In constructing our MIMO system, the base of the exponent in the edge weight decay function was derived by considering the median of scores for all cell types generated by the model. Using a model-trained value could potentially lead to significant variations in the interaction values between every pair of cell types. Hence, to prevent excessive elimination of edges for any particular cell type, we opted for a fixed value that is moderate and applicable across all cell types.


$$ corr\left({f}_b^{(a)},{t}_c^{(a)}\right)=\frac{f_b^{(a)}{t}_c^{(a)} / \left({z}^{(a)}-1\right)}{\sqrt{\operatorname{var}\left({f}_b^{(a)}\right)}\sqrt{\operatorname{var}\left({t}_c^{(a)}\right)}}, $$

$$ corr\left({u}_{\mathrm{h}}^{(a)},{l}_c^{(a)}\right)=\frac{u_b^{(a)}{l}_c^{(a)} / \left({z}^{(a)}-1\right)}{\sqrt{\operatorname{var}\left({u}_b^{(a)}\right)}\sqrt{\operatorname{var}\left({l}_c^{(a)}\right)}}, $$

In the equation, *corr*() and *var*() calculate the Pearson correlation coefficient and variance, respectively. ${f}_b^{(a)}$ and ${u}_{\mathrm{h}}^{(a)}$ represent the preprocessed scores of neighboring cell type *b* and the preprocessed expression values of HVG *h* in cell type *a*, while${t}_c^{(a)}$ and ${l}_c^{(a)}$ are the scores of the *c*-th block obtained during CPPLS regression modeling. These *P*-values are adjusted using the Benjamini–Hochberg (BH) method [37] to obtain the false discovery rate (FDR)–adjusted values ${s}_{b,c}^{(a)}$and ${s}_{h,c}^{(a)}$. A parameter *δ* is set, and if *δ* is greater than or equal to 0.05, the coefficients ${w}_{b,h}^{\prime (a)}=\sum_c{w}_{b,h,c}^{\prime (a)}$are returned:


$$ \left\{\begin{array}{@{}ll}{w}_{b,h,c}^{\prime (a)}={w}_{b,h,c}^{(a)}\ & if\ {s}_{b,c}^{(a)}<\mathrm{\delta} <{\mathrm{s}}_{\mathrm{h},\mathrm{c}}^{\left(\mathrm{a}\right)}<\mathrm{\delta}, \\{}{w}_{b,h,c}^{\prime (a)}=0\ & otherwise,\end{array}\right. $$

The second filtering step involves CPPLS calculating the *P*-values ${s}_{h,c}^{\prime \prime (a)}$ for the genes that were not filtered out in the first step but lack statistical significance using the BH method. For coefficients ${w}_{b,h}^{\prime (a)}$ of each cell type *a*, if *δ* is greater than or equal to 0.05, the coefficients ${w}_{b,h}^{\prime \prime (a)}=\sum_c{w}_{b,h,c}^{\prime (a)}$are returned:


$$ \left\{\begin{array}{@{}c}{w}_{b,h}^{\prime \prime (a)}=\kern0.5em {w}_{b,h,}^{\prime (a)}\kern8em if\kern0.75em {s}_{b,h}^{\prime (a)}<\mathrm{\delta} ,\\{}{w}_{b,h}^{\prime \prime (a)}=\kern0.75em 0\kern10em otherwise .\end{array}\right. $$

### Clustering of HVGs

Before clustering, all the HVGs with coefficients equal to 0 are filtered out. For each cell type *a*, CPPLS-MLP employs the *k*-means method to cluster the filtered coefficients of HVGs [38]. The optimal number of clusters *k* is determined using the Silhouette method. *k* ranges from the minimum integer value 2 to the maximum integer value 15. we utilize Silhouette analysis to determine the number of clusters for each cell type clustering, ranging from 2 to 15. Silhouette analysis assesses the quality of clustering results by considering both the compactness and separation of clusters. For each data point, a Silhouette coefficient is computed, ranging from −1 to 1, with values closer to 1 indicating better clustering results. By trying different values of *k* and calculating the average Silhouette coefficient for each *k* value, we can identify the optimal range of *k* values.

### The construction of MIMO graph

CPPLS-MLP will summarize the two-point diagram obtained in the previous step:


$$ {w}_{a,b}=\sum_2^{15}\sqrt[2]{{\left({w}_{b,i}\right)}^2\dots +{\left({w}_{b,j}\right)}^2}\kern0.75em ,\left(2\le i\le j\le 15\right)\kern0.5em , $$

In the equation, ${w}_{a,b}$ represents the square root of the coefficients of gene clusters within neighboring cell type *b* for cell type *a*. The aggregated coefficient matrix W is summarized by calculating its mean. Values below the mean are set to 0, and the remaining values are visualized in the form of a directed graph, illustrating the MIMO interactions between cells. In constructing the MIMO system from bipartite graphs, we ensure that the number of gene clusters within each cell type does not influence the final decision by standardizing the gene cluster counts across all cell types to their minimum value.

To align with the principle that the homeostasis within the tissue is collectively maintained by all cells, and considering that communication weakens as the distance between cells increases, CPPLS-MLP assumes pathways such as A → B, A → C → B, A → C → D → B (where A, B, C and D represent distinct cell types). Moreover, CPPLS-MLP acknowledges that the strength of communication exponentially decreases as the number of intermediate cells along the path increases. Therefore, based on the obtained coefficient matrix W, CPPLS-MLP calculates pathways from cell type *a* to all other cell types and applies attenuation to each path *k*:


\begin{align*}& {weight}_{a,b,k}=\left({weight}_{a,i}- mean\ast{10}^{1-i}\right)+\dots +\\&\left({weight}_{a,j}- mean\ast{10}^{1-j}\right)\kern0.5em ,\kern0.75em \left(0\le j\le i\le \mathrm{D}\right) \end{align*}

*j*, *i* represent the first side and the last side of the departure from *a* → *b*, respectively. The path of the final *a* → *b* is:


$$ {weight}_{a,b,k}=\sum_0^{+\infty }{weight}_{a,b, ki},\left(0\le i\le +\infty \right)\kern0.5em . $$

Getting the path matric matrix R of all cell types can be visually displayed in the form of chord diagram.

### Identification and classification of genes involved in cell communication

CPPLS-MLP initially filters the top 2000 HVGs using the FindVariableFeatures() method [39]. In addition to this, CPPLS-MLP employs three other methods, namely, variance [40], scranpy [41] and M3drop [42], to select 16 000 HVGs across both datasets. These 16 000 HVGs are categorized into two classes based on predefined labels from databases such as GeneCards and Gene Ontology (GO). In our single-cell transcriptomics analysis, we selected the top 2000 HVGs related to cell communication as our analysis threshold because the default threshold of the FindVariableFeatures() method is 2000, and we did not alter it. Moreover, limiting the number of variable genes significantly enhances computational efficiency when processing large datasets, thereby avoiding unnecessary resource consumption. Therefore, adopting the top 2000 HVGs as our threshold is a result of our comprehensive consideration of multiple factors, aimed at improving the efficiency of constructing cell communication networks.

(i) Strong correlation(strong_com): ‘receptor’, ‘ligand’, ‘receptors’, ‘ligands’, ‘cell–cell adhesion’, ‘intercellular interaction’.
(ii) Weak correlation(weak_com): ‘surfaces of many cells and extracellular matrices’, ‘Participates in cellular’, ‘Pathway’, ‘regulation’, ‘signal transduction’.

During manual querying, genes are assessed for their relevance to cell communication. A strong correlation is used as the criterion: genes with a label are assigned a value of 1, while those without are given 0.

In the process of cell communication, interactions often occur in conjunction with LRIs. Therefore, prior to tagging, we reviewed information recorded in the GeneCards and GO databases for 16 000 HVGs, extracting a total of 11 tags including ‘receptor’, ‘ligand’, ‘receptors’, ‘ligands’, ‘cell–cell adhesion’ and ‘intercellular interaction’, among others. We considered these tags directly related to the cell communication process as strongly relevant. For other tags that describe gene functions related to cell communication, we categorized them as weakly relevant. The MLP possesses powerful non-linear mapping capabilities and has been widely applied in bioinformatics for classification tasks [43, 44]. Therefore, CPPLS-MLP utilizes the genes selected through FindVariableFeatures() from both datasets and employs the MLP neural network model to classify these genes.

## RESULTS

### Overview of the CPPLS-MLP method

**Figure 1:**
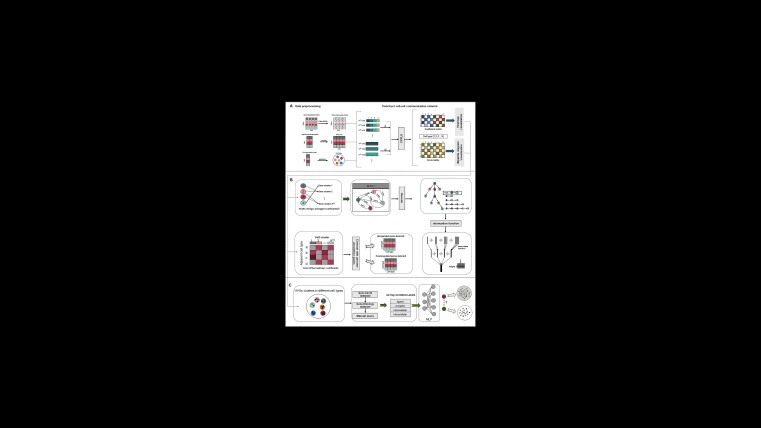
Workflow of the CPPLS-MLP method and visualization. (**A**) CPPLS-MLP processes gene expression and spatial information from single-cell and ST datasets. Subsequently, the data are modeled using the CPPLS model for subsequent analysis. (**B**) Cell–cell communication strength is influenced by proximity. In the MIMO system, all paths between each pair of nodes in the graph are extracted. Edge weights are attenuated based on the number of nodes along the paths. This process yields the optimal weights and bipartite graphs between the two cell types, visualized as directed graphs and heat maps. (**C**) Gene clusters involved in constructing the cell communication network are analyzed. Strong relevant labels are extracted from GeneCards and GO databases. Using the neural network model MLP, these clusters are classified based on their association with cell communication.

Figure 1 provides an overview of the workflow developed and tested for CPPLS-MLP, primarily comprising two main components: (1) construction of cell–cell communication networks based on single-cell and spatial transcriptomic data, including gene expression matrices and spatial coordinate matrices, and (2) identification and classification of gene clusters involved in network construction. In the first part, utilizing single-cell gene expression matrices, spatial coordinate matrices from ST data and cell type label data, a linear model (CPPLS) is employed to capture the multi-response (continuous, discrete and combined) relationship between intercellular communication and HVGs expression (Figure 1A). This step constructs the cell communication network, wherein edge weights are attenuated based on the number of nodes along the paths, yielding the final communication strength between two cell types (Figure 1B). By integrating the BH method and Silhouette method, the model utilizes 10-fold cross-validation to obtain average metrics, generating a coefficient matrix representing the direction and degree of regulation for each HVG’s expression by adjacent cell types.

The second component of CPPLS-MLP involves labeling and categorizing HVGs that contribute to constructing the cell communication network (Figure 1C). Utilizing four methods, FindVariableFeatures(), variance, scannpy and M3drop, 16 000 HVGs were selected through manual queries in official databases like Genecard and GO. Strong relevant labels, identified as strong_com, were extracted. These labels were used to tag the HVGs involved in CPPLS-MLP modeling. Subsequently, a neural network model, MLP, was employed to classify these genes.

CPPLS-MLP is also employed for visualizing cell–cell communication networks and assessing the extent and direction of HVG expression modulation by intercellular communication (Figure 1B). For example, in single-cell datasets, it evaluates relationships between gene clusters and neighboring cells, as well as interactions among different cell types. This analysis and visualization utilize two distinct spatial techniques and their corresponding datasets: the seqFISH+ mouse cortex dataset and the Seq-Scope mouse colon dataset.

### Performance comparison of CPPLS-MLP with other methods

The construction of the cell–cell communication network by CPPLS-MLP serves as the foundation for subsequent analyses. To evaluate its performance, two single-cell and ST datasets from mouse cortex and mouse colon were utilized. CPPLS-MLP demonstrated its ability to identify known HVGs whose expression is influenced by intercellular communication in spatial contexts, as shown in cases based on the seqFISH+ mouse cortex and Seq-Scope mouse colon datasets. Although the number of predicted HVGs regulated by cell–cell communication varied across methods, a significant overlap was observed between CPPLS-MLP and other approaches. This suggests the reproducibility of inferences made by these methods.

Therefore, we proceeded to conduct a horizontal comparison, evaluating CPPLS-MLP’s performance in inferring HVGs expression regulated by intercellular communication. Additionally, we compared its gene classification capabilities with those of existing methods. CPPLS-MLP consistently outperformed these methods across the benchmark datasets, securing the top position. Illustrative examples from the seqFISH+ dataset, depicting interactions between neural layers, indicated that CPPLS-MLP might be more effective in single-cell and ST datasets characterized by higher the uniqueness of HVG among different cell types.

**Figure 2:**
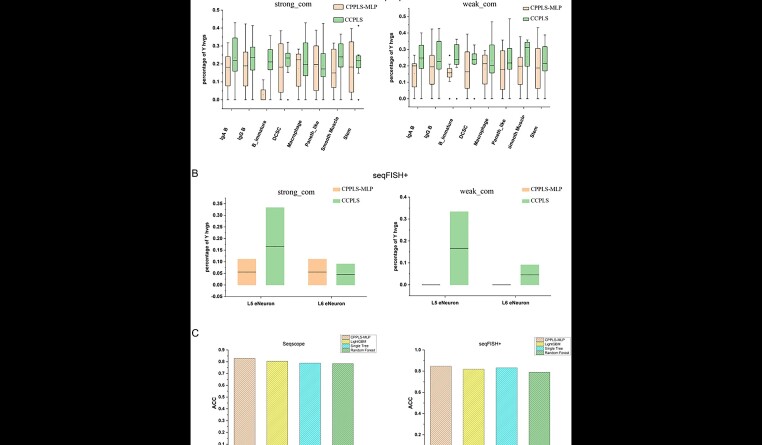
Superior performance of CPPLS-MLP compared to existing methods. (**A**) Comparison of the uniqueness of Y HVGs in each cell type based on strong and weak correlation labels between CPPLS-MLP and existing methods estimating intercellular communication-regulated HVG expression (CCPLS) in the SeqScope dataset. (**B**) Comparison of the uniqueness of Y HVGs in each cell type based on strong and weak correlation labels between CPPLS-MLP and CCPLS in the seqFISH+ dataset. (**C**) Performance comparison of CPPLS-MLP with some existing classification methods (LightGBM, Single Tree and Random Forest) on the Seq-Scope and seqFISH+ datasets.

Because CPPLS-MLP is not limited to estimating cell–cell communication solely based on LRIs for regulating HVGs in intercellular differential expression, we conducted a comparative analysis with other existing methods, specifically CCPLS, which estimate the degree and direction of HVG regulation through intercellular communication. We ensured uniformity in cell types across the comparison datasets. In both Seq-Scope and seqFISH+ datasets, we categorized the HVGs involved in cell communication networks into communication-related genes (Y HVGs) and non-related genes (N HVGs) based on strong and weak correlation labels. After calculating the percentage of Y HVGs within each cell type cluster in relation to the total genes obtained, we determined the overlap rate of Y HVGs between different cell types. As depicted in Figure 2A and B, on the Seq-Scope dataset, CPPLS-MLP demonstrated higher uniqueness of HVGs in only two cell types, Macrophage and Paneth_like, compared to CCPLS under strong correlation labels. Additionally, under weak correlation labels, CPPLS-MLP showed slightly lower uniqueness than CCPLS specifically in macrophage cells. In the seqFISH+ dataset, under strong correlation labels, HVGs in L6.eNeuron cells exhibited slightly higher uniqueness with CPPLS-MLP compared to CCPLS. This difference was due to the fact that CCPLS identified only three genes as Y HVGs in this specific cell type. Under weak correlation labels, CPPLS-MLP achieved a Y HVGs overlap rate of 0 in these two cell types. This result provides evidence that the HVGs identified by CPPLS-MLP in constructing cell communication networks are more representative for each specific cell type.

Subsequently, when labeling HVGs based on strong correlation labels, we compared the performance of CPPLS-MLP with existing classification methods. We posited that genes associated with cell communication follow specific expression patterns, enabling their classification through methods trained on labeled data. As illustrated in Figure 2C, CPPLS-MLP exhibited superior performance on both datasets, outperforming several existing classification methods. In summary, these results indicate that CPPLS-MLP is a relatively accurate and effective approach for deducing the regulation of intercellular communication on HVGs’ intercellular differential expression and for classifying HVGs based on cell communication labels.

We compared our method with popular approaches like cellphoneDB and Cellchat by considering the top 50% of their predicted results and evaluated the number of genes related to cell communication identified by each method based on strong correlation labels. In the seqFISH+ mouse brain dataset and the Seqscope human colon dataset, our CPPLS-MLP method identified 354 and 345 genes related to cell communication, respectively. In contrast, cellphoneDB identified 284 and 104 genes, respectively, for the seqFISH+ and Seqscope datasets, while Cellchat identified 388 and 142 genes, respectively.

### Identification of signals between fibroblasts and B.cell_IgA cells

**Figure 3:**
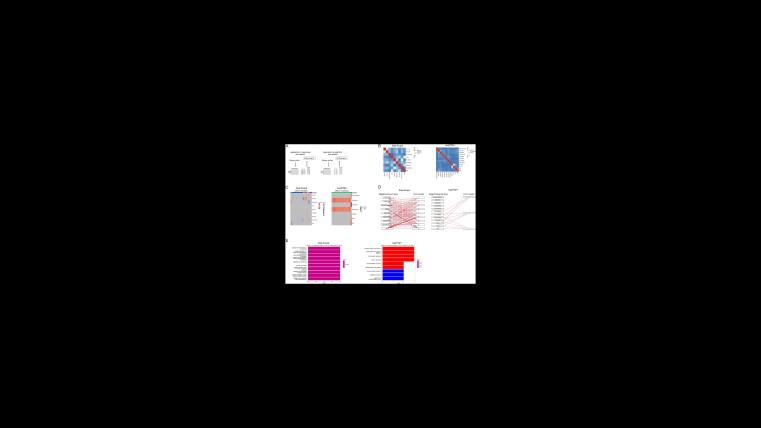
Single-cell and ST real data sets Seq-Scope and seqFISH+ are applied to CPPLS-MLP. (**A**) seq-scope and seqfish+dataset schematic diagram. (**B**) The overlap rate of HVGs between different cell types is at two real data concentrations. (**C**) A heat map was generated to display the relationship coefficients between HVG clusters within fibroblast cells and other cell types in the Seq-Scope real dataset experiment, as well as between HVG clusters within L5.eNeuron cells and other cell types in the seqFISH+ real dataset experiment. (**D**) Bipartite graph showing the relationship between gene clusters and adjacent cell types in B_immature cells and L5.eNeuron cells. (**E**) GO enrichment of genes in B.cell_lgA cells and L5.eNeuron cells.

First, we applied CPPLS-MLP to the single-cell and ST dataset, Seq-Scope mouse colon dataset (Figure 3A left). This dataset encompasses 10 806 genes and nine distinct cell types: B immature cells, DCSC, IgA B cells, macrophages, smooth muscle cells, stem cells, Paneth-like cells, fibroblasts and IgG B cells. Before utilizing CPPLS-MLP, we examined the HVGs extracted among the nine cell types in the real Seq-Scope dataset. The experimental findings revealed that the average overlap ratio of HVGs between these cell types was 0.31 (Figure 3B left). Specifically, most highly variable genes differed across different cell types, indicating unique characteristics for HVGs in each cell type. Next, we clustered the HVGs within each cell type to infer their relationships with other cell types. Visualization was performed using heat maps and bipartite graphs (Figure 3C and D left; Supplementary Figures S1 and S2). Additionally, we conducted GO enrichment analysis on the detected gene clusters (Figure 3E left; Supplementary Figure S3).

**Figure 4:**
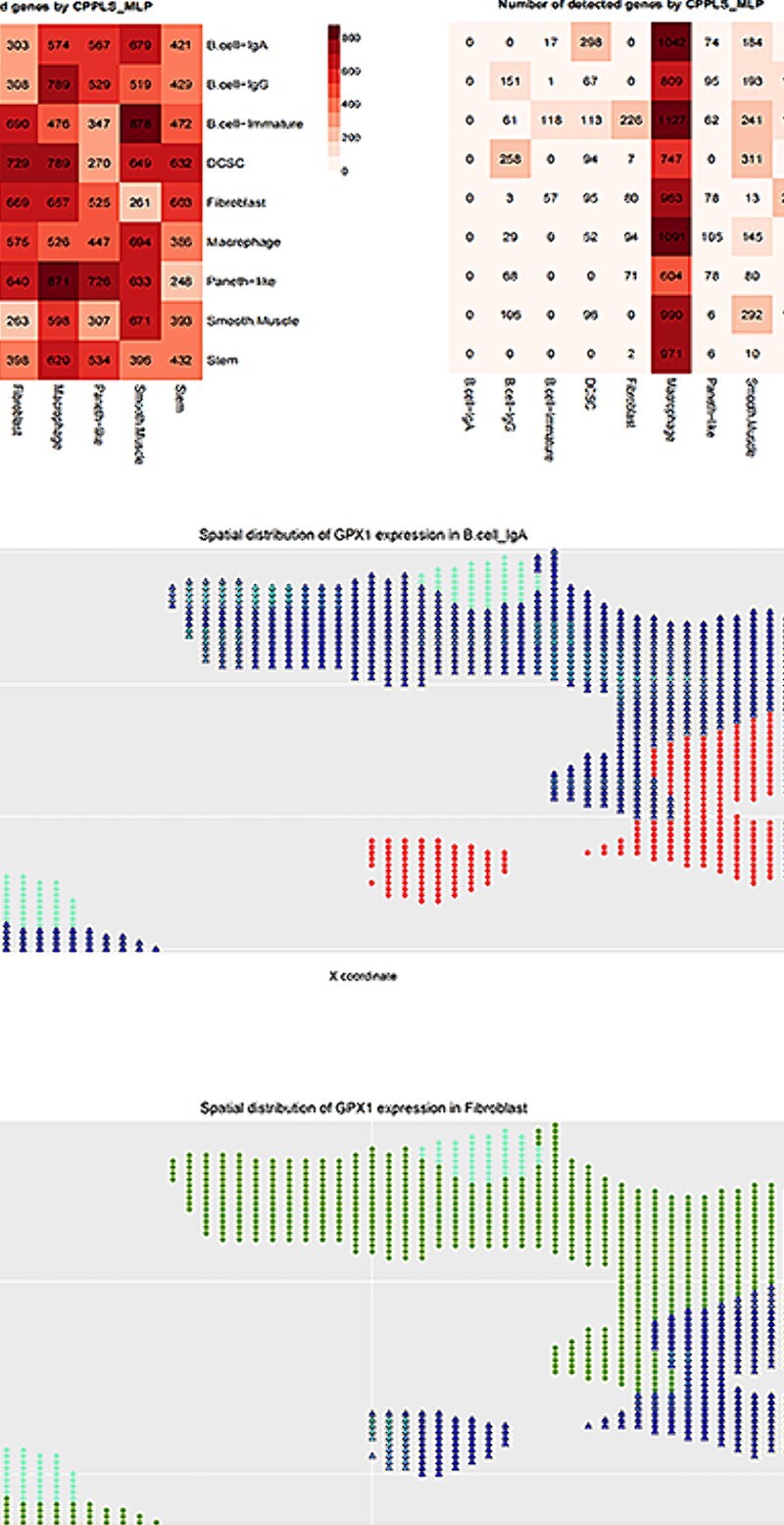
Identification of signals between fibroblasts and B.cell_IgA cells. (**A**) Upregulated genes detected by CPPLS-MLP in the Seq-Scope dataset. (**B**) Downregulated genes detected by CPPLS-MLP in the Seq-Scope dataset. (**C**, **D**) The two-dimensional distribution diagrams of gene GPX1 expression in cell types B.cell_IgA and fibroblast, respectively.

CPPLS-MLP was employed to assess the impact of intercellular communication on HVG expression by comparing the detected genes with the average expression levels in the mouse and human genome annotation packages (Figure 4A and B). Further exploration focused on the communication between immature B cells and fibroblasts. Fibroblasts, known as cancer-associated fibroblasts (CAFs), constitute a major stromal component in cancer and play a crucial role in maintaining tissue homeostasis by interacting with molecules within the extracellular matrix (ECM). B.cell_IgA cells, on the other hand, have vital roles in the immune system. These two cell types influence each other’s states and functions through molecular interactions in the ECM, contributing to the stability of colonic tissue. Both B cells and fibroblasts secrete cytokines such as IL-6, IL-8 and IL-10, which regulate immune responses [45]. Notably, fibroblasts not only serve as microenvironmental regulators of intestinal stem cells but also modulate lymphatic endothelial cells, blood endothelial cells and immune cells in the intestine. Through signaling interactions with different signal molecules and cell types, fibroblasts play crucial roles in intestinal development, homeostasis and diseases [46]. In cases of intestinal diseases, abnormal ECM secretion by fibroblasts may disrupt the regulation of epithelial cells, leading to pathological fibrosis. Hence, fibroblasts secrete ECM to interact with immune cells, regulating inflammation and immune responses to help control tissue inflammation processes.

Taking the gene GPX1 as an example, the protein encoded by this gene belongs to the glutathione peroxidase family, catalyzing the reduction of organic hydroperoxides and hydrogen peroxide by glutathione [47]. It exerts antioxidative effects in fibroblasts, reducing the impact of oxygen radicals. In communication with B.cell_IgA cells, GPX1 mitigates oxidative stress on B.cell_IgA cells through its antioxidative properties. We further plotted the positions of cells in a two-dimensional space and visualized the expression levels of the GPX1 gene in B.cell_IgA cells and fibroblasts (Figure 4C and D). It is noteworthy that the GPX1 gene is known to be associated with colitis, indicating its involvement in the regulation of inflammatory signaling pathways. Additionally, in GO analysis, the GPX1 gene is annotated with ‘epithelial cell development’. Notably, at the boundary between B.cell_IgA cells and fibroblasts, B.cell_IgA cells exhibited high expression of the GPX1 gene. These findings suggest that the epithelial cell development in B.cell_IgA cells occurs through their interactions with fibroblasts. The MIMO interactions in the Seq-Scope real data set are illustrated in a directional graph (Supplementary Figure S8A).

Finally, CPLS-MLP adopts the neural network model MLP to classify the top 2000 HVGs with the largest expression differences between cells, which are extracted using the FindVariableFeatures() method and labeled according to the strong correlation labels selected from the dataset.

### Recognition of preference communication in the fifth and sixth layers of mouse brain neurons

Next, CPPLS-MLP was applied to study and visualize intercellular communication within the mouse cortex dataset from the single-cell and ST dataset, seqFISH+. This dataset comprised data from 10 000 sequenced genes, covering 12 cell types, including Adarb2 iNeuron, astrocytes, endothelial cells, L2/3 eNeuron, L4 eNeuron, L5 eNeuron, L6 eNeuron, Lhx6 iNeuron, microglia, mural cells, oligodendrocytes (Olig) and oligodendrocyte progenitor cells (OPCs) (Figure 3A right). Before applying CPPLS-MLP, we examined HVGs extracted from interactions between these 12 cell types in the seqFISH+ real dataset. In our experiments, the average overlap ratio of HVGs between these cell types was 0.25 (Figure 3B right), indicating the uniqueness of cell-type-specific sets of highly variable genes across different cell types. Subsequently, HVGs from each cell type were clustered to infer their relationships with other cell types. The relationships were visualized using heat maps and bipartite graphs (Figure 3C and D right; Supplementary Figures S4 and S5). Additionally, the detected gene clusters were subjected to GO enrichment analysis (Figure 3E right; Supplementary Figure S6).

CPPLS-MLP initially examined the impact of intercellular communication on the expression of HVGs in the seqFISH+ dataset (Supplementary Figure S7A). It identified communication between cells L5 eNeuron and L6 eNeuron, both belonging to the pyramidal neurons of the mouse brain’s fifth and sixth cortical layers, respectively. Neurons of this type typically transmit information in neural networks [48]. Experimental evidence has shown that in the visual and somatosensory cortex, the intracortical axons of L6 CT neurons primarily target L5a. When activated, L6 CT neurons trigger action potentials in L5a pyramidal neurons. Additionally, the activation of L6 CT neurons inhibits the excitatory neurons in L4 [49]. This interaction pattern plays a crucial role in visual and somatosensory processing.

For example, CPLX1 in L5 eNeuron belongs to the synaptic protein gene family, encoding a protein involved in synaptic vesicle exocytosis. Its protein product binds to the SNAP receptor complex and disrupts it, allowing neurotransmitter release. These processes play a crucial role in communication between the neurons in the brain’s neural cortex and regulate bodily movements [50]. CPPLS-MLP further plotted a two-dimensional spatial distribution of L5 eNeuron and L6 eNeuron. It was observed that the closer L5 eNeuron is to L6 eNeuron, the higher the expression level of CPLX1 in L5 eNeuron, indicating that the communication between L5 eNeuron and L6 eNeuron influences the expression of HVGs within the cells (Supplementary Figure S7B). The directional graph illustrating the MIMO in the seqFISH+ real dataset is provided in Supplementary Figure S8B.

Finally, CPPLS-MLP employed the neural network model MLP to classify the top 2000 HVGs exhibiting the most significant intercellular expression differences, as determined by the Find VariableFeatures() method and based on strong correlation labels, within this dataset.

## DISCUSSION

We have demonstrated the impact of cell–cell communication on HVGs expression and its accurate classification using CPPLS-MLP in two single-cell and ST datasets, Seq-scope and seqFISH+. These datasets encompass single-cell and ST data generated by Seq-Scope and seqFISH+ methods.

When applying the Seqscope dataset, we aimed to predict communication between immature B cells and fibroblasts. Communication between these two cell types occurs via the extracellular matrix and plays a crucial role in maintaining the homeostasis of colon tissue. To validate our predictions, we visualized the expression levels of the GPX1 gene in both cell types and its spatial distribution. This visualization confirmed the accuracy of our predictions. When applying the seqFISH+ dataset, we aimed to predict communication between L5 eNeurons and L6 eNeurons. These two cell types communicate via neurons and play a crucial role in maintaining visual and somatosensory processing. To confirm our predictions, we visualized the expression levels of the CPLX1 gene and its spatial distribution in these two cell types. This visualization confirmed the accuracy of our predictions.

For assessing model performance, we indeed selected two single-cell and ST datasets, namely, from the mouse cortex and human colon. Despite the limited number of datasets, we chose these due to their representativeness, covering various organs and cell types. Additionally, we believe these two datasets align well with the application scenario of our model, as cellular communication in tissues like the cortex and colon is crucial for understanding their biological functions. There are two principles explaining how cell–cell communication impacts the expression of HVGs and allows for classification: adjacent cells can communicate through extracellular signaling molecules (such as growth factors, cytokines, hormones, etc.), which can influence the gene expression patterns of HVGs cells, leading to differential expression; genes from two interacting cells have similarities in their expression profiles. This hypothesis is grounded in several theoretical and empirical studies, such as the Correlation AnalyzeR, a method for functional gene prediction based on the analysis of gene co-expression correlations. Existing scRNA-seq technologies, which identify cell subpopulations associated with specific phenotypes based on single-cell data, have been widely applied to determine cell types and states, revealing significant differences in gene expression profiles across different cell types. However, we have also observed that within similar biological environments, such as the same tissue type or neighboring cell groups, there can be a degree of similarity in gene expression profiles between cells. This similarity may reflect interactions and communication between cells, including the interaction of cytokines and the activation of signaling pathways. Therefore, ST data are particularly suitable for inferring cell–cell communication-regulated differential expression of HVGs based on these principles and can be classified using specific strategies. In this context, our proposed method CPPLS-MLP combines the cell–cell communication network and labels genes in a known database to filter out HVGs that are not involved in cell communication. Then, it utilizes the multivariate linear regression properties of CPPLS to model the spatial coordinates of cells and gene expression information. Finally, it classifies genes based on strong correlation labels. Thus, on benchmark ST datasets, the uniqueness and accuracy of classification for the HVGs involved in CPPLS-MLP modeling surpass existing methods, demonstrating the applicability of these principles in decoding cell–cell cross-talk. This approach is especially suited for deciphering the regulation of cell–cell communication on HVGs expression.

Furthermore, CPPLS-MLP infers that interacting cells exhibit similarity in their gene expression profiles, indicating that genes within interacting cells are governed by specific expression patterns. By manually querying genes in databases like Genecard and GO and extracting labels, CPPLS-MLP offers novel insights into assessing whether genes are associated with cellular communication. Currently, analyzing and visualizing ligand–receptor pairs in scRNA-seq data at single-cell resolution is challenging. CPPLS-MLP, on the other hand, analyzes the relevance of genes to cell communication based on gene expression patterns and labels. If certain genes within ligands and receptors exhibit similar expression patterns, the cells harboring these genes are likely engaging in communication. CPPLS-MLP integrates spatial coordinates, gene expression data and extracted strong and weak correlation labels, offering a new perspective on evaluating intercellular communication.

In the inference of cell–cell communication, incorporating spatial information and gene categorization is crucial for spatial analysis of intercellular communication. Utilizing prior knowledge to classify genes as relevant or irrelevant to cellular communication aids in computational inference of intercellular communication. Furthermore, in studying spatially regulated cell–cell communication, integrating different omics data and multimodal datasets such as 10x Multiome and Digital Spatial Profiling provides more comprehensive, multidimensional information. We propose utilizing image processing techniques to extract precise cell location information from tissue slice images in ST data. This location data will provide us with accurate information about cell positions within tissues, enriching the data foundation for our research. By integrating this spatial information, we can overcome the challenge of analyzing data when only gene expression is available. This enhancement will not only make CPPLS-MLP compatible with current multimodal data but also improve our ability to explore biologically meaningful cell–cell interactions. This approach enhances our understanding of cell interactions and regulatory mechanisms. In this context, reliable computational models are needed to accurately integrate multimodal data and perform inference.

**Key Points**

- We developed an innovative model integrating neural networks and multiple linear regression functions, called CPPLS_MLP. This model uses coordinate information in spatial transcriptomic data and gene expression information in single-cell sequencing data to accurately construct a cell communication network and classify HVGs in the network based on existing database information.
- CPPLS_MLP exhibits excellent accuracy and robustness, making it suitable for various experimental designs, single-cell sequencing presumably derived from different organs and cell communication of ST data.
- CPPLS_MLP is provided in an open-source format and can be directly used to predict cell communication in different tissues and classify genes in their communication networks.

## Supplementary Material

CPPLS-MLP_Supplemental_Material_bbae198

## Data Availability

For seqFISH+，the single-cell ST data of the mouse somatosensory cortex dataset was retrieved from the “Github repository [https://rubd.github.io/Giotto_site/articles/mouse_seqFISH_cortex_200914]” [25].For Seq-Scope, the single-cell ST data of the human colon dataset was downloaded from “Deep Blue Data[Data Set | Seq-Scope processed datasets for liver and colon results (RDS) and H&E images | ID: 9c67wn05f | Deep Blue Data (umich.edu)]” [51]. Source codes for the CPPLS_MLP R, python packages and the related scripts are available at “CPPLS_MLP Github [https://github.com/wuzhenao/CPPLS-MLP]”.

## Author Biographies

**Tianjiao Zhang** is an associate professor of College of Computer and Control Engineering at Northeast Forestry University of China. His research interests include bioinformatics.

**Zhenao Wu** is a master candidate of College of Computer and Control Engineering at Northeast Forestry University of China. His research interests include bioinformatics.

**Liangyu Li** is a master candidate of College of Computer and Control Engineering at Northeast Forestry University of China. His research interests include bioinformatics.

**Jixiang Ren** is a master candidate of College of Computer and Control Engineering at Northeast Forestry University of China. His research interests include bioinformatics.

**Ziheng Zhang** is a master candidate of College of Computer and Control Engineering at Northeast Forestry University of China. His research interests include bioinformatics.

**Guohua Wang** is a professor of College of Computer and Control Engineering at Northeast Forestry University of China. He is also professor of Faculty of Computing at Harbin Institute of Technology of China. His research interests are bioinformatics, machine learning and algorithm.
