# Supplementary material for: CPPLS-MLP: a method for constructing cell–cell communication networks and identifying related highly variable genes based on single-cell sequencing and spatial transcriptomics data
Source: Brief Bioinform. 2024 Apr 27;25(3):bbae198. doi: 10.1093/bib/bbae198 (PMC11056015; doi:10.1093/bib/bbae198)
Supplement: CPPLS-MLP_Supplemental_Material_bbae198 [file cppls-mlp_supplemental_material_bbae198.pdf]

Supplementary Materials

Figure S1. Heat map of all the cell types in the Seq-Scope real dataset.

Figure S2. Bipartite graph of all the cell types in the Seq-Scope real dataset.

Figure S3. Gene Ontology (GO) enrichment of all the cell types in the Seq-Scope real dataset.

Figure S4. Heat map of all the cell types in the seqFISH+ real dataset.

Figure S5. Bipartite graph of all the cell types in the seqFISH+ real dataset.

Figure S6. Gene Ontology (GO) enrichment of all the cell types in the seqFISH+ real dataset.

Figure S7. Detection of genetic effects and 2D visualization of CPLX1 gene expression in seqFISH+ real dataset.

Figure S8. The directed graph of MIMO is shown in Seq-Scope and seqFISH+ real datasets.

<u>Apllication to the Seq-Scope real dataset:</u>

Color of heatmap: coefficient

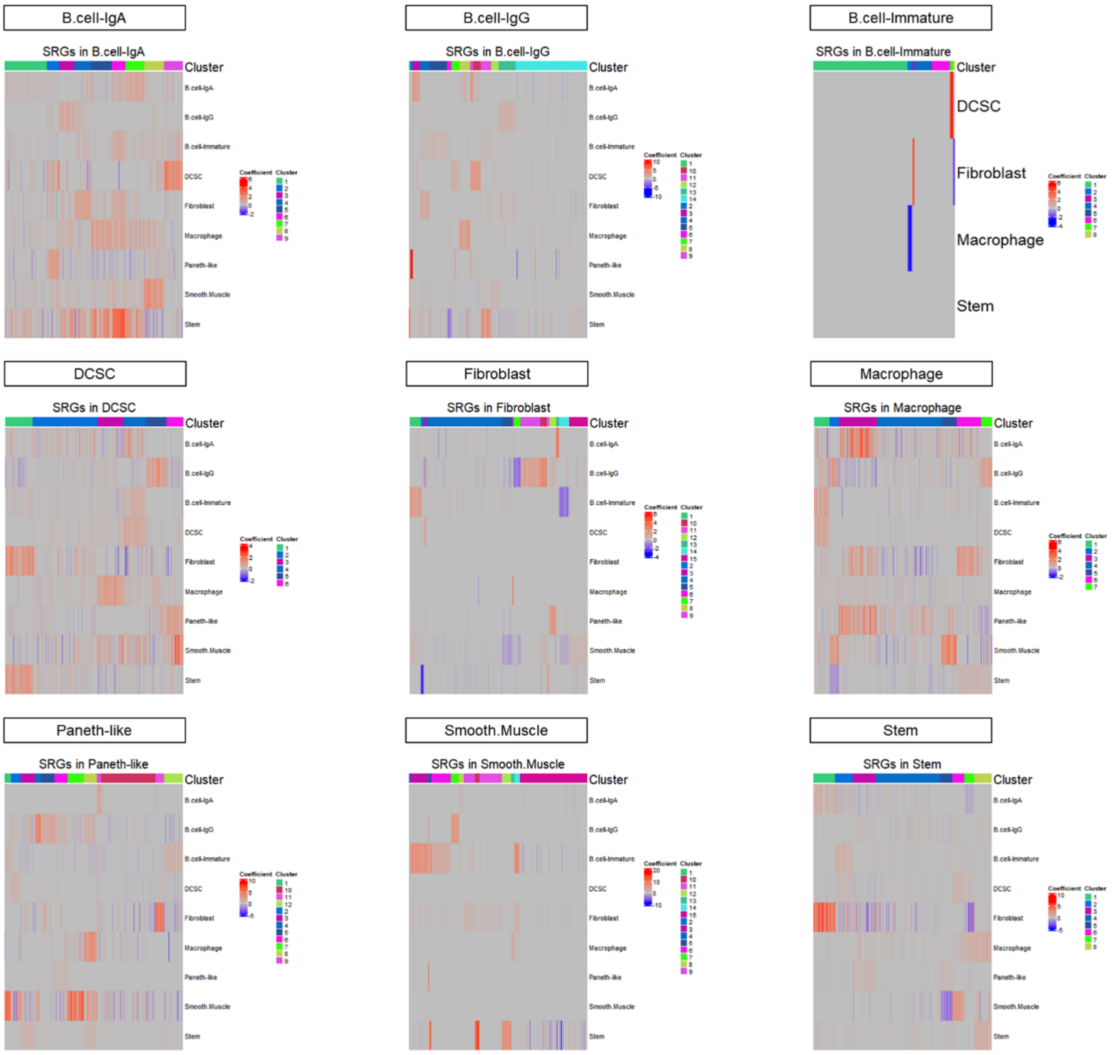

Figure S1. Heat map of all the cell types in the Seq-Scope real dataset. Rows and columns correspond to neighboring cell types and highly variable genes (HVGs), respectively. The color of the heat map indicates the coefficient.

<u>Apllication to the Seq-Scope real dataset:</u>

Width of edge: averaged coefficient/5

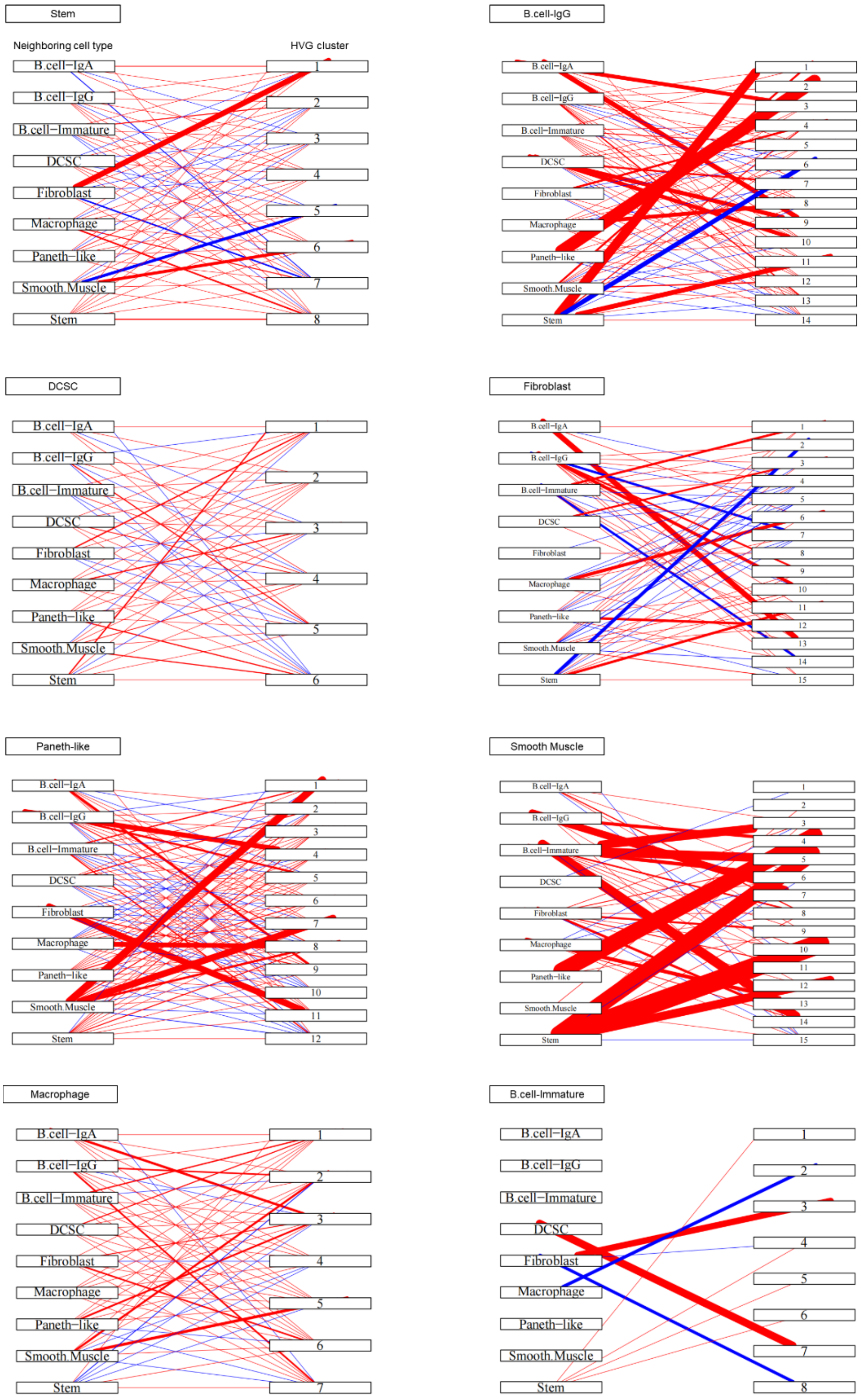

Figure S2. Bipartite graph of all the cell types in the Seq-Scope real dataset. The width of each edge indicates the averaged coefficients/5 in each combination of highly variable gene (HVG) clusters and neighboring cell types.

<u>Apllication to the Seq-Scope real dataset:</u>

Row: GO term Column: gene count

Color of bar graph: adjusted p-value

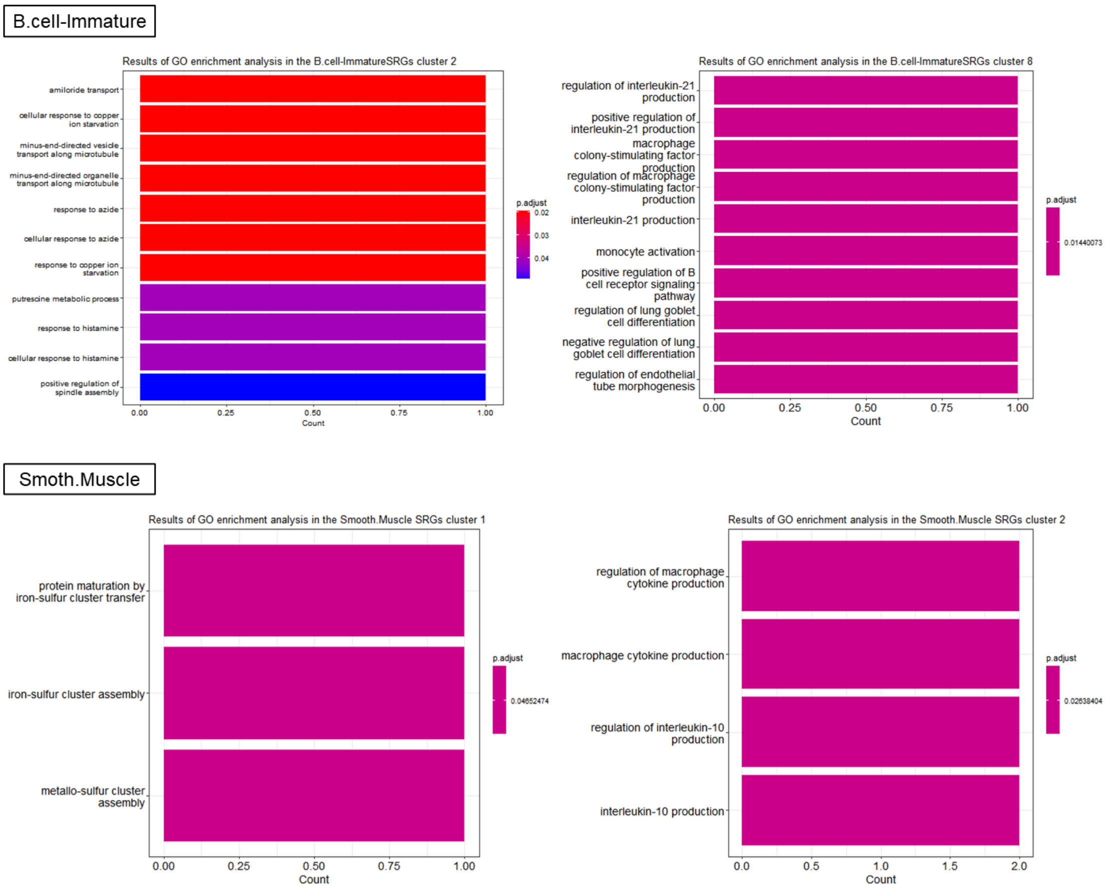

Figure S3. Gene Ontology (GO) enrichment of all the cell types in the Seq-Scope real dataset.

Apllication to the seqFISH+ real dataset:

Color of heatmap: coefficient

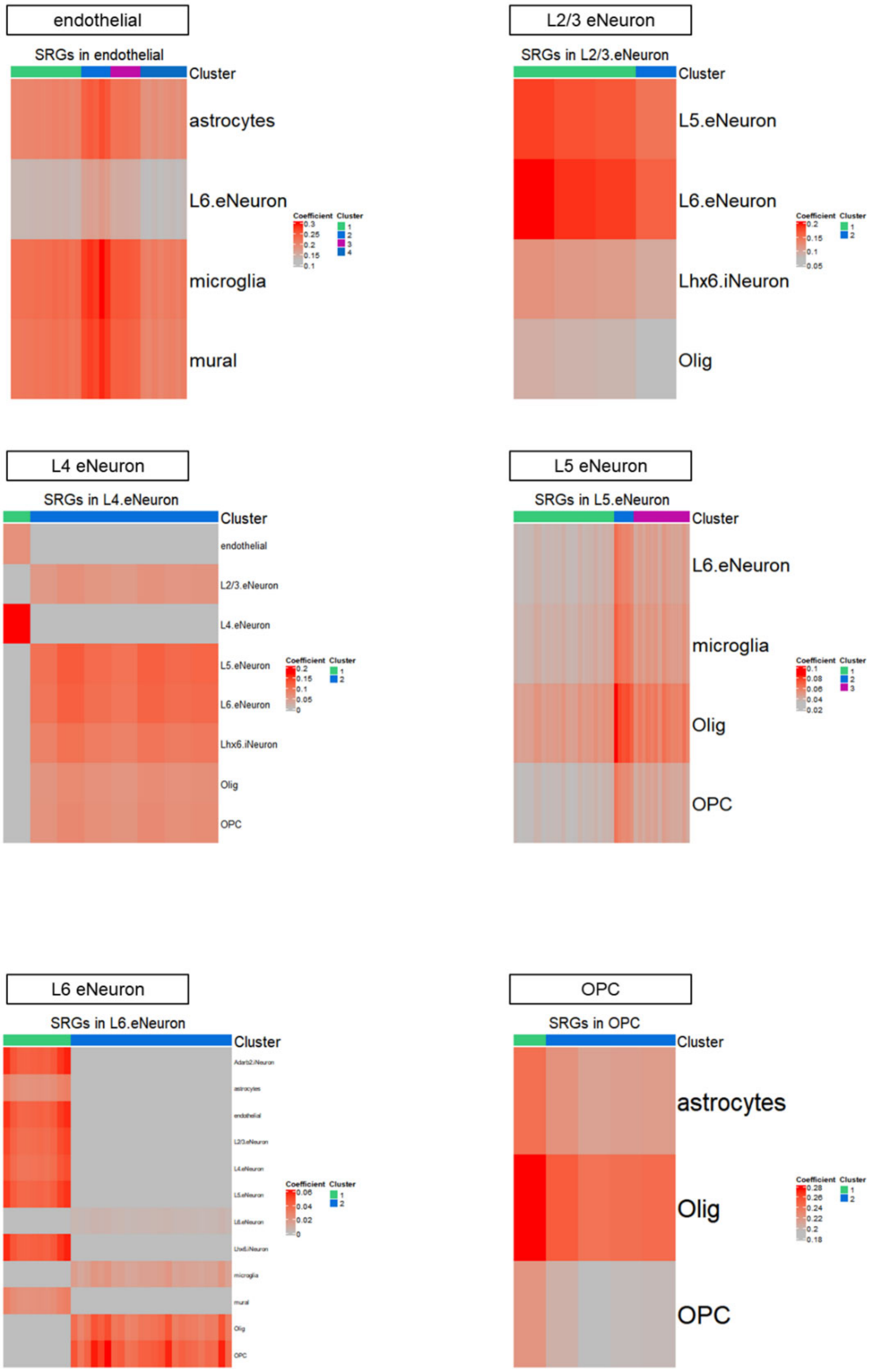

Figure S4. Heat map of all the cell types in the seqFISH+ real dataset. Rows and columns correspond to neighboring cell types and highly variable genes (HVGs), respectively. The color of the heat map indicates the coefficient.

Apllication to the seqFISH+ real dataset:

Width of edge: averaged coefficient/5

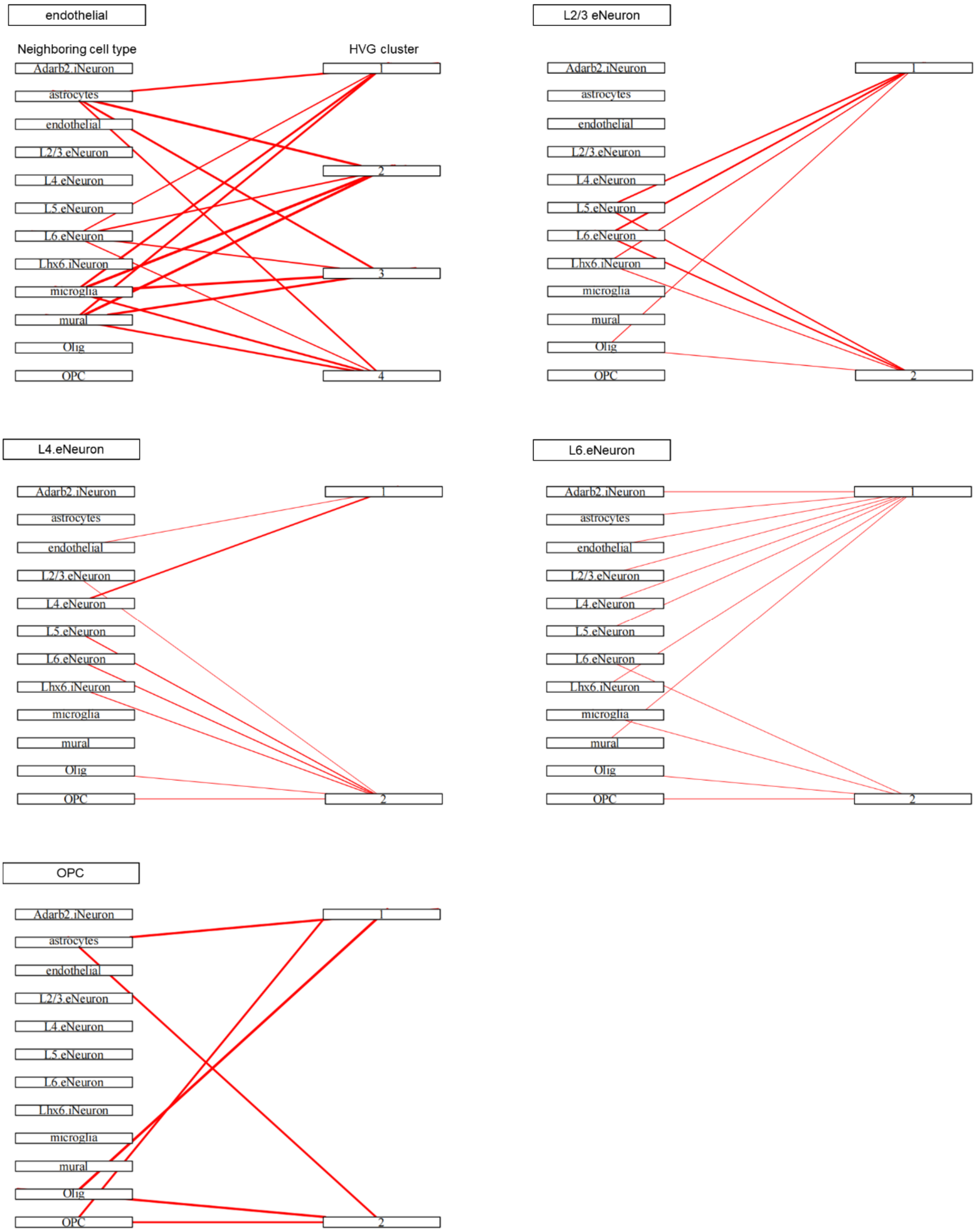

Figure S5. Bipartite graph of all the cell types in the seqFISH+ real dataset. The width of each edge indicates the averaged coefficients/5 in each combination of highly variable gene (HVG) clusters and neighboring cell types.

<u>Apllication to the seqFISH+ real dataset:</u>

Row: GO term Column: gene count

Color of bar graph: adjusted p-value

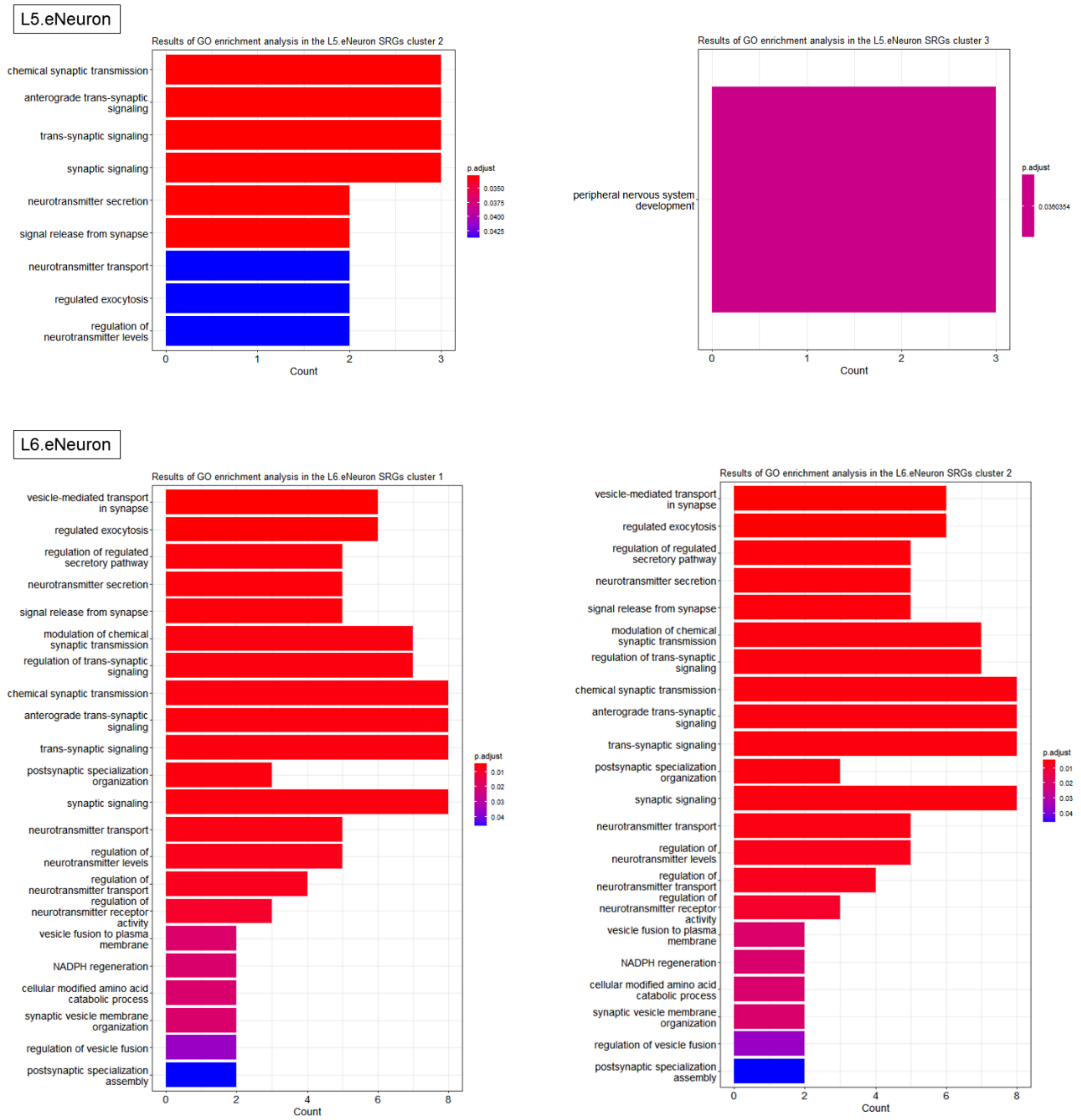

Figure S6. Gene Ontology (GO) enrichment of all the cell types in the seqFISH+ real dataset.

Apllication to the seqFISH+ real dataset:

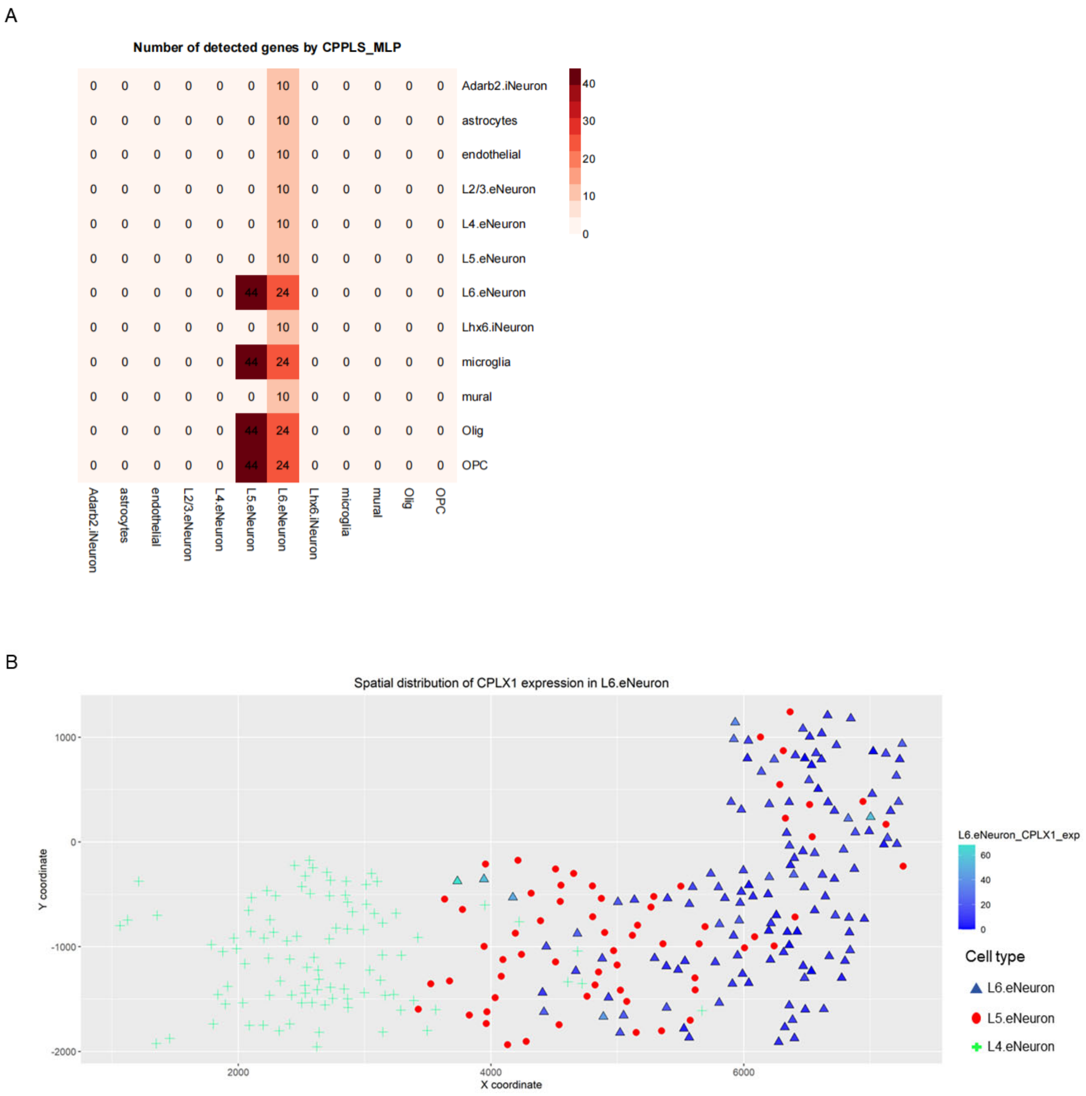

Figure S7. (A) Upregulated genes detected by CPPLS-MLP on the real dataset of seqFISH+ data. (B) Expression distribution of gene CLPX1 in L6.eNeuron cells.

Apllication to the Seq-Scope and seqFISH+ real dataset:

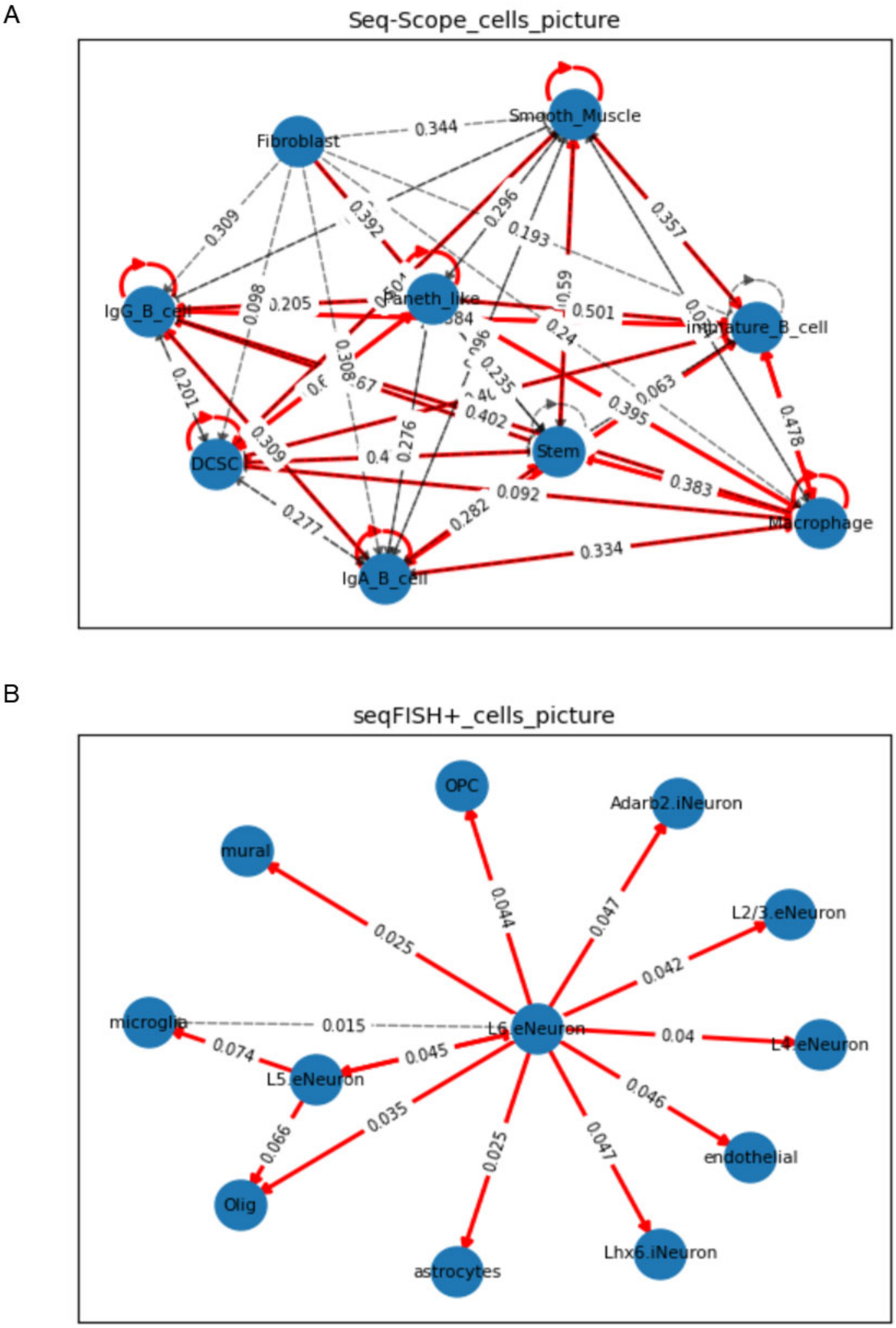

Figure S8. (A) MIMO of communication between different cell types obtained by applying CPPLS-MLP to the real dataset of Seq-Scope data. (B) CPPLS-MLP is applied to MIMO of communication between different cell types obtained from the seqFISH+ real data set.
